# A striving for independence: a qualitative study of women living with vertebral fracture

**DOI:** 10.1186/1472-6955-9-7

**Published:** 2010-04-16

**Authors:** Inger Hallberg, Anna-Christina Ek, Göran Toss, Margareta Bachrach-Lindström

**Affiliations:** 1 Department of Medical and Health Sciences, Division of Nursing Science, Faculty of Health Sciences, Linköping University, SE-581 85 Linköping, Sweden; 2 Department of Medical and Health Sciences, Division of Cardiovascular Medicine/Internal Medicine, Faculty of Health Sciences, Linköping University, SE-581 85 Linköping, Sweden; 3 Department of Endocrinology & Gastroenterology/Osteoporosis Unit, University Hospital, SE-581 85 Linköping, Sweden

## Abstract

**Background:**

Quantitative studies using generic and disease-specific health-related quality of life (HRQOL) questionnaires have shown that osteoporosis-related vertebral fractures have a significant negative effect on HRQOL, but there are only few studies that address what it means to live with vertebral fracture from a deeper experiential perspective. How HRQOL and daily life are affected several years after vertebral fracture and how women cope with this are more unclear. This study aimed to describe how HRQOL and daily life had been affected in women with vertebral fracture several years after diagnosis.

**Methods:**

The study design was qualitative. Semi-structured interviews were conducted with ten Swedish women during 2008. Data were analysed using qualitative inductive content analysis.

**Results:**

The findings of this study revealed three themes related to the influence on HRQOL and daily life: *A threatened independence*, i.e. back pain, anxiety, negative impact on self-image and consequences in daily life; *Strategies for maintaining independence*, i.e. coping, self-care and support; and *The importance of maintaining independence*, i.e. the ability to perform everyday activities, social interaction and having something meaningful to do. The women were striving for independence or maintaining their independence by trying to manage different types of symptoms and consequences in different ways.

**Conclusion:**

HRQOL and daily life were strongly affected in a negative way by the impact of the vertebral fracture. Information from this study may provide new knowledge and understanding of the women's experiences of living with vertebral fracture from an insider's point of view in order to obtain a deeper understanding of the women's everyday life. However, further evaluation is still needed in larger study groups.

## Background

Vertebral fracture resulting from osteoporosis is very common, and is the most frequent osteoporotic fracture in developed countries [1,2]. Osteoporosis is defined as a systematic skeletal disease characterized by low bone mass and the microarchitectural deterioration of bone tissue, with a consequent increase in bone fragility and susceptibility to fracture [3]. Osteoporosis causes no symptoms besides fractures and their complications. Osteoporosis-related fractures, particularly of the vertebrae and hip, may lead to impaired health-related quality of life (HRQOL) [4-8].

During recent decades, cross-sectional studies [9-11] and some follow-up studies [12-14] after vertebral fracture have reported that HRQOL is severely impaired. Few studies examine what it means to live with a vertebral fracture; one describes the experience of five women with vertebral fractures, with each participant describing significant challenges in maintaining daily functioning [15]. Two studies focus on how self-concept provides an understanding of the range of strategies women with osteoporosis use to manage their chronic illness in daily life [16,17].

The consequences of vertebral fracture can be grouped into three categories: pain, physical changes and impairment, and psychosocial declines [18].

There is a need to describe and understand the impact of vertebral fractures on older women's HRQOL and daily life, to be able to provide more supportive health care for these women. This was revealed by new aspects gathered from an open-ended question following the SF-36 in a seven-year follow-up survey study (unpublished data) that had not appeared in the previous quantitative studies. However, the long-term impact of vertebral fracture on HRQOL and daily life has not been evaluated sufficiently. The aim was to describe how health-related quality of life and daily life had been affected in women with vertebral fracture several years after diagnosis.

## Methods

A inductive conventional approach of content analysis was used [19,20]. The advantage of this approach is that it makes it possible to obtain direct information from study participants without predetermined categories or theoretical perspectives [19]. The interview gives participants an opportunity to describe in their own words their experiences in detail and give their perspectives and interpretations. This method accesses the participants' understanding of their real life and experiences [21].

### Participants and setting

Study participants were originally recruited from a group of 303 Swedish women [14,22] who, two years earlier, had completed a seven-year follow-up study after a vertebral or hip fracture [23]. Out of these 303 women, 51 were possible for inclusion in this study. From this group a purposeful sampling of ten women with experiences of living with vertebral fracture and a strategic sampling to achieve maximal variation on dimensions of interest were chosen [24]. Variations were sought by age, marital status and number and severity of vertebral fractures and other previous osteoporosis-related fractures.

The women were aged between 68 and 84, and all lived at their own residence, nine women in independent housing and one woman in senior housing. Half of the women were married and half were widowed. The women had had an average of four vertebral fractures and a severity of 11 according to the Spinal Deformity Index (SDI) [25]. No women had any indication of new vertebral fracture the previous six months. Overall, nine women reported one or more co-morbidity conditions, the most frequent being cardiac disease, rheumatic or musculoskeletal and bronchi-pulmonary disorders. Nine women were undergoing current pharmacological treatment for osteoporosis, and most used painkillers regularly or sometimes (Table 1).

**Table 1:** Participant demographic details

Interview	Age	Marital status	Children	Number of Vertebral Fractures *	SDI *	Other Fracture/s Yes/No
1	74	Widow	Yes	7	15	No
2	84	Widow	Yes	4	10	Yes
3	80	Married	Yes	6	16	Yes
4	79	Married	Yes	3	7	Yes
5	72	Married	Yes	4	9	Yes
6	82	Widow	Yes	7	18	Yes
7	68	Married	Yes	5	11	No
8	79	Widow	Yes	3	8	No
9	70	Widow	No	5	14	Yes
10	79	Married	Yes	1	3	No

*Number and grade of vertebral deformities were assessed two years before the present study by evaluating a lateral digital radiograph of the thoracic and lumbar spine, according the Genant visual semi-quantitative criteria. A Spinal Deformity Index (SDI, range 0-39) was calculated as the sum of all deformity grades, from 0 (normal) to 3 (severe) in each of the thoracic and lumbar vertebrae from T4 to L4, a high score indicating a more severe diagnosis.

### Interviews

Interviews were conducted by the first author (IH) and lasted from 29 to 69 minutes, excluding the informal conservation that took place before and after the interview to build contact and allow women to ask questions. The data collection was performed in the women's homes (n = 7) or at the first author's office (n = 3), according to the women's preference. The data were collected from April to November 2008.

A semi-structured interview guide was used [24]. Interviews were digitally recorded (MP3, Philips digital voice tracer 7890) and transcribed verbatim, by one of the authors (IH) and a professional secretary, including any nonverbal or background sounds. A transcription guide was used [26].

The central question was: Could you tell me how your quality of life and daily life have been affected by the vertebral fracture? Further topics were: How do you cope with your symptoms after the vertebral fracture? What is most important to you, what matters most in life? What kinds of support would make your daily life easier? The interviewer posed probing questions in order to deepen, clarify and develop the women's responses. Examples of probing questions in the study were "How did you feel?" and "What did you think?"

### Analysis

The analysis was inspired by Hsieh and Shannon [19]. The conventional content analysis consisted of the following steps:

1. Transcripts were checked for accuracy.

2. The analysis started with a reading from beginning to end of all the transcripts by the authors independently.

3. Each transcript was read carefully, word by word, and the text that appeared to be relevant to the aim was highlighted by the first author.

4. The texts were broken down into phrases, using the participants' words (keywords or statements that are related to each other based on their content and context), which were then condensed. The label of the condensed phrase was referred to a preliminary code and was related to the comprehensive content of the phrase. Nonverbal sounds, pauses and filler words such as "hm" supported the interpretation. This analysis was performed by the first author, and then all the authors took part in the interpretations and labelled the phrases as codes.

5. After open coding of four transcripts, preliminary codes and a coding scheme was decided. The remaining transcripts were coded and the original ones recoded, using these codes and adding new ones when the data did not fit into an existing code.

6. When all transcripts had been coded the first author grouped the codes, according to how they were related, which were then agreed on by the other authors. Some codes were combined during this process.

7. The final step was to implement the coding process in all transcripts and organize them into a hierarchical structure in the form of subcategories and categories. The various subcategories were compared in terms of similarities and differences. Subcategories with similar content were grouped together and preliminary categories were formulated. The analysis process involved continuous movement between the whole and the parts of the text. Finally, themes were formulated from the underlying meaning of the categories.

### Ethics

The women were provided with written and oral information regarding voluntary participation and explaining that their responses would be confidential. The women gave oral informed consent beforehand and written at the visit. They were also informed that the digitally recorded interview would be transcribed and that their names would be given specific code numbers to ensure confidentiality. The study was approved by the Regional Ethical Review Board at the Faculty of Health Sciences, University of Linköping 2005, registration no. M173-05, and was performed in accordance with the Declaration of Helsinki [27].

## Results

**Figure 1:**
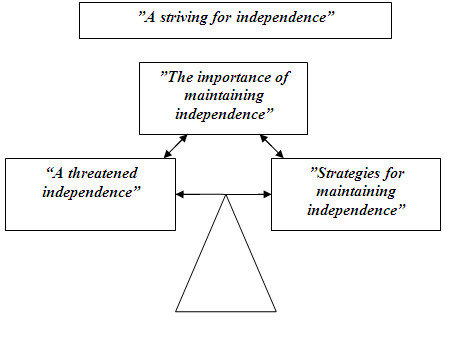
**A striving for independence**. The women with vertebral fracture in this study are striving for balance in daily life by trying to manage different types of symptoms and consequences in different ways in order to maintain their independence.

The findings revealed three themes related to how vertebral fractures affect HRQOL and daily life: *A threatened independence*, *Strategies for maintaining independence *and *The importance of maintaining independence*. The women with vertebral fracture were striving for balance in daily life by trying to manage different types of symptoms and consequences in different ways in order to maintain their independence (Figure 1). The categories and subcategories within each theme are displayed in Table 2.

**Table 2:** Subcategories, categories and themes

Subcategories	Categories	Themes
-Constant back pain	-Back pain	**A threatened independence**
-Activity-related back pain		
-The body as a hindrance in everyday life	-Consequences in daily life	
-Dependent on others		
-Feelings of loneliness	-Anxiety	
-Fear and threat		
-Self-esteem	-Self-image	
-Loss of roles		
-Bodily change		
-------------------------------	-------------------------	---------------------------------
-Optimistic coping	-Emotional	**Strategies for maintaining independence**
-Pessimistic coping		
-Self-care	-Activity	
-Social support	-Support	
-Health care professional support		
-----------------------------------	--------------------------	---------------------------------
-Ability to perform everyday activities	-"Managing"	**The importance of maintaining independence**
-Experience health		
-Next of kin and friends	-"Loving"	
-Social activities		
-Living conditions	-"Having"	
-Something meaningful to do		

### A threatened independence

The first theme describes how HRQOL and daily life have been affected by the vertebral fractures through back pain, anxiety, consequences in daily life and a negative impact on self-image (Figure 1, Table 2).

#### Back pain

The statements about back pain were described in two subcategories, *Constant back pain *and *Activity-related back pain*, as very central in their lives.

Many women described the **constant back pain **as terrible/horrible and as a totally unnatural life-dominating pain that you never forget. Back pain was described as prolonged pain, a very special kind of pain, that affected one's whole life:

"It was a long-lasting and serious pain that really affected...my whole life...and it also lasted a long time...it didn't fully go away like other pains for a long time...you forget other pains, unlike this unnatural pain."

It appeared that the dull back pain was constant and could suddenly get worse. This experience led to a total change into being, as one woman describes it, "like a zombie". The pain was experienced as constant, and when it became too much it was hard to keep one's mood up.

**Activity-related back pain **was described as a pain that comes on suddenly during specific physical activities and makes continued activity impossible. For example, it may appear in activities such as walking, carrying, bending or travelling. When the activity stops, the pain eases or ceases. The women also described difficulties in finding a comfortable position in bed and in chairs due to back pain.

"I remember it as completely dominating, stabbing as well as dull, but particularly these very intense stabs...when you moved from one position to the other."

Tiredness in the back was described as feelings of discomfort that lead to the ongoing activity being interrupted or adjusted. This discomfort could sometimes be relieved by certain adjustments of movement or aids that support the back, but in some cases the ongoing activity must be stopped completely. Similar problems could also be described as lumbar weakness when a woman's back was not holding her body upright.

#### Anxiety

The anxiety category was divided into two subcategories: *Feelings of loneliness *and *Fear and threat*.

Many women experienced **feelings of loneliness **or worry about being alone. Back pain and the inability to go upstairs or sit down in an ordinary chair led to an avoidance of getting together with relatives and friends. It was also found that some women felt like a burden to others, and that they were not social or friendly enough.

"You lose the desire...it's just like that...when you're feeling...no I can't cope with this...I don't want to...because I can't do it...it's depressing, it actually is."

**The fear and threat **of further back pain, falling and fracture were common.

When the threat of back pain and uncertainty appear, one's daily life is affected:

"And then this horrible back pain comes...and then I'm completely lost...I feel like I almost just want to collapse, and I have to either sit or lie down."

One of the most commonly expressed concerns was a fear of falling and suffering a new fracture. Several women worried about becoming dependent if they suffered a new fracture. They became cautious or avoided several activities, for example walking outside or travelling. This fear was experienced as a threat, a handicap affecting daily life.

#### Consequences in daily life

Consequences in daily life were described in two subcategories: *The body as a hindrance in everyday life *and *Dependent on others*.

The women described how living with vertebral fracture forced them to make changes in several areas in their life, of greater or smaller importance. They had to adjust their daily activities to a slower, more careful, or reduced rate. Some women had completely stopped certain activities, such as cooking, cleaning or shopping. Being affected by vertebral fracture could be described as a sudden disruption of and change in everyday life - **the body as a hindrance in everyday life**. Decreased physical function and back pain were the most frequently expressed symptoms affecting their daily life negatively. That the handicap was not visible could cause situations to be experienced as even worse. One woman expressed that the pain and complications from the vertebral fracture hindered her from, as she put it, "living".

"...so of course it has affected the whole situation of life...you're not mobile in the same way...you can't go, get to anywhere...do anything."

Women expressed difficulties with the change in life situation because they were **dependent on others **in coping with everyday life. They were irritated and sorry that they needed help from their husband, children or friends to be able to move or manage housework, leisure and social activities. Always needing help affected one's mood negatively. The sense of feeling like a burden was also something that emerged in the interviews.

"...and can't manage on my own...you've been used to always having done that...that you're dependent on others."

#### Self-image

Self-image was described in: *Self-esteem*, *Loss of roles *and *Bodily change*.

**Self-esteem **was influenced by the woman's view of herself and by interaction with others. They did not feel like they were treated with respect by people like health care professionals since their back pain and other symptoms were invisible.

"Sometimes I get a notion that they think I'm...acting...that it's not as difficult as it is."

Experiences of feeling left out and not feeling sociable or nice enough in interaction with others emerged.

**Loss of roles, **for example not being able to manage one's home, affected the women's self-image. They did as much as they could, but wanted to do more:

"My husband had to help me out a lot with normal chores...because he had to cook, he had to do everything...grocery shopping.I just sat there like some...some sort of package on a chair and watched."

An experience of **bodily change **was expressed as not being able to keep one's body erect. The women used words such as shrunken, pocketknife, bent and stooped. They were concerned about their changing body and how it would be in the future. A threat to the women's self-image and quality of life was described:

"I've shrunk even more, it affects...it affects my quality of life...because I'm quite vain, so I want to look good..."

The sense of feeling old and that of being old were associated with the changing body shape and back pain. The need to ask for help, assistance and aids such as a walking frame also enhanced the feelings of being old:

"I don't think I'm that old...but I obviously am, so I should be using one of those walking frames...because I do need it...but - ah - it's embarrassing."

They described a sense of being old as growing old prematurely and being a shrunken old woman. It was the body that was old, while the feeling inside was the same.

### Strategies for maintaining independence

The second theme describes how the women were trying to manage their situation to maintain independence through various emotional and activity strategies and support (Figure 1, Table 2).

#### Emotional

The women described how, with different emotional strategies, they tried to manage their suffering and discomfort. Two subcategories were given: *Optimistic coping *and *Pessimistic coping*.

**Optimistic coping **entailed that the women tried to make the best of the situation and to have a positive attitude. One way to master the situation was to think of something else, that it could have been worse. Another way was to not focus on the back pain, but accept it by living here and now. The women tried to think positively to see the good side of the situation. One way to handle the situation was also to simply accept it:

"It would be really great to not have this pain...and all the lumbago...but you just have to try to live with it...I don't know of anything else that works...so you have to try to make the best of the situation ...and find something positive...for instance, baking...I think that makes it a little...positive"

When the women accepted the situation and organized their life practically by avoiding certain situations according to their symptoms, they experienced well-being.

**Pessimistic coping **entailed having a negative attitude about the situation and sometimes expecting the worst to happen. It revealed feelings of hopelessness and situations being difficult to master. Living day by day was a common strategy. One woman described it as nothing being able to be done to improve it:

"No...I don't know what I should have done...no, no,...I can't think of anything...that I should have done to get better, ease or anything...there isn't a lot of help to get..."

#### Activity

The women used activity-related strategies to obtain relief from suffering and discomfort. One of the most common **self-care **strategies was taking pain killers. They also described trying to balance between effect and side effect of the painkillers as well as a fear of addiction.

Another self-care strategy was to rest, lying or sitting down. The women also had strategies for lying down in bed and getting up. The consequences after the vertebral fracture had caused adjustments in the women's daily routines, making them slow down or give up housework. Exercise was acknowledged, as strength and balance improved in such a way that other activities in daily life became easier to perform. But adhering to a regime of daily regular physical exercise was reported as difficult due to a lack of motivation and knowledge, as well as tiredness in the back. Types of exercise reported were walking, self-training programmes and aquatic exercise as well as everyday activities at home. A fear of becoming dependent and unable to live at home can be seen as a motivating factor for continuing to exercise.

"Ending up in an institution is what terrifies me ...I'm scared to death of ending up somewhere where I can't manage on my own...and maybe in a way that's a good thing...it makes me stronger to be able to...feel that I need (to exercise)..."

The women used different aids to provide relief from back pain or discomfort such as heating pad, massage, motorized bed or chair with adjustable backrest. To make physical exercise and daily activities easier, they used: cane, walking poles, slip-resistant material on shoes and walking frame. The use of a walking frame helped keep the body upright and allowed the women to rest when necessary.

#### Support

Support has two subcategories, *Social support *and *Health care professionals' support*.

Next of kin and friends play an important role in the women's life, providing both emotional and instrumental **social support**. Married women perceived their husbands as most supportive when they assisted in the household and gave emotional support. Children and especially grandchildren meant a great deal, especially when they visited or called, which gave the women energy and strength. Many women received practical assistance from their children:

"They saw how bad it was, so they helped me...and when I went shopping they always came with me and carried...the bags...because they don't think I should strain my back..."

Being able to help or support someone else emerged as important for the women's own well-being. Relational problems and lack of time, understanding and emotional support were common complaints by the women who described their next of kin and friends as unsupportive.

Regarding **support from health care professionals**, most women expressed both positive and negative experiences of support they had received. When health care professionals made an effort to listen, understand and provide support and treatment, the women experienced that they were treated with understanding and respect. The same was experienced when they received answers about the fracture, treatment for the osteoporosis or back pain, and help with the exercise programme. Knowing you could get help when you needed it was very important. Not being treated with understanding or knowledge, as well as being ignored, was commonly experienced.

"They (health care professionals) say that...well, you're just going to have to see yourself like this and live with it...that makes you a bit pessimistic sometimes...and it makes you feel...abandoned..."

Most women felt that the help they received consisted largely of medicine and radiological examinations. Some received back pain relief from pain killers, but most believed that they had not received adequate treatment.

Women stressed the importance of learning what to do and what not to do. Many were aware of the importance of exercise, but needed support and help concerning what they could do. They were afraid of doing something wrong, and did not dare exercise.

It was important to have confidence in the health care professionals.

### The importance of maintaining independence

The third theme describes what the women felt were the most important values in life and can be described with the following categories: Managing, Loving and Having (Figure 1, Table 2).

#### Managing

The women described how they strive to take care of themselves and manage everyday life through the subcategories *Ability to perform everyday activities *and *Experienced health*. Managing daily life and the **ability to perform everyday activities **were very important to them all. Managing one's home, feeling free and being able to move about freely and do what one wants was perceived as quality of life.

"Yeah, do things on my own and like...get up and eat breakfast...that's worth a lot...I don't do much...I do what's most important...fix food, do the wash and take care of myself."

The women described that they **experienced health **if they could manage on their own and take care of themselves, and not feel dependent on others. Being able to move about freely and do what one wants was very central and important to the women's experience of health and quality of life.

"That I can move around, that I can be outside and walk...if I weren't able to do that...it would be awful...so I guess it's that that's...my wish...to be able to go outside and walk...being outside is quality of life...and being able to keep living here and not need to...have help from others but instead manage on my own."

Managing everyday life can be understood with the meaning of "managing", being able to take care of oneself and to have an influence and the opportunity to influence one's own life and be able to realize one's vital goals in life.

#### Loving

Good relationships, social interactions and the ability to participate in social activities, such as doing things with next of kin or friends, emerged as very essential. *Next of kin and friends *and *Social activities *are the subcategories in this category.

One woman described the importance of good relationships and interactions with **next of kin and friends **as follows:

"Laughing together, that's very important...that's quality of life...you could say that...and being able to get together and have good friends...and not sit there alone...the whole day and not see anyone."

Children and especially grandchildren meant a great deal, and many women looked forward to seeing them. Women also stated that **social activities **are important and central to independent living. Participating in social and cultural activities, leaving the house when they wanted to go to a restaurant or society/club were seen as important for feeling independent. Social interaction can be understood as "loving", which satisfies the need for human relationships.

#### Having

This category can be described by the subcategories *Living conditions *and *Something meaningful to do*.

It appeared that the **living conditions**, such as the home, dwelling and surrounding environment, were of great importance. The garden, flowers, or just being able to sit on the balcony and enjoy the outdoor nature was important as well.

Having **something meaningful to do **such as solving crosswords, knitting or doing needlework was also important.

"I love my home and want to keep busy...and I think it gives me a lot, that it's...often my salvation that I have...something to do...and can feel like it gives me a bit."

"Having" is about material needs like housing and something meaningful to do as well as the associated factors.

## Discussion

The aim of this study was to describe how HRQOL and daily life had been affected in women with vertebral fracture several years after diagnosis.

The study showed that women's daily lives were strongly influenced by the impact of their vertebral fracture, even several years after diagnosis. As a result of how their independence and daily life had been affected by the fractures, the women in this study were striving for HRQOL by trying to manage different types of symptoms and consequences through different ways of maintaining their independence. The importance of maintaining independence and autonomy was reiterated throughout the interviews. The women had a strong volition to manage on their own, which caused them to adapt their activities in several ways (Figure 1).

Vertebral fracture usually receives less attention from health care professionals, next of kin, friends and society than do fractures in the forearm or hip, because its symptoms are invisible. It is also supposed that the pain ceases after only a few months [28]. This study demonstrates the opposite: despite the passing of several years between diagnosis and interview, the women still complained about pain.

Health-related quality of life or health status is a subset of quality of life, concerning physical, emotional, and social well-being [4,29]. The results from the previous quantitative study demonstrated that women with vertebral fracture had remaining pronounced reduction of HRQOL regarding the SF-36 domains at seven-year follow-up [23]. The current study also shows new aspects of the meaning of HRQOL and daily life for these women with vertebral fracture. The study shows that the constant and activity-related back pain had a severe impact on the women's ability to manage daily life that threatened their independence. A study of elderly women with vertebral fracture suggested that constant pain and perceived lack of control had a severe impact on the women's ability to perform daily activities [15]. It was suggested in a previous study that physical change and functional limitations, including the inability to carry out normal activities and participate in social activities, may influence the loss of self-esteem most directly [8]. This corresponds with the results in this study, but the loss of social roles also had an impact on self-image.

A fear and threat of falling were common in this study. A recent study concluded that the greatest negative effect on HRQOL was associated with self-reported fear of falling [30].

The loss of the housekeeper role may be especially difficult for this age group of women, who traditionally view the home as their domain. Not being able to manage the home was perceived as difficult and affected the women's self-image as well as their independence. This finding is supported by a study focusing on the role of the family and service community [31].

A qualitative study of 28 elderly women with osteoporosis concluded that the women used diverse strategies to manage their osteoporosis on a day-to-day basis depending on their self-concept [16]. Women with confident selves regarded aging and chronic illness as manageable, accepted their limitations and could master changes in their lives. Women with contradictory selves were struggling to gain control over their lives and denial was their predominant strategy. Women with disparaged selves talked about themselves with a lack of self-respect and self-worth, and preferred a resignation strategy [16]. In the current study, women with higher self-esteem seem to manage their situation better than women with lower self-esteem did. An awareness of these strategies may be useful in understanding how women with vertebral fracture manage their situation and make decisions to seek care from health care professionals.

The results show similarities as well as differences between the women in the group. The women who expressed a positive self-image mentioned a wide range of useful strategies for managing their daily life. These women seemed to manage their situation better than did those with a more negative self-image. They also expressed a lower degree of threatened independence. Women who used strategies to maintain their independence in the form of optimistic coping, the ability to perform active self-care, and social support from the environment expressed a sense of balance in life. On the other hand, those with more pessimistic coping and a lower ability to perform self-care and who experienced decreased social support from next of kin and/or friends perceived a threatened independence in the form of more back pain, anxiety, consequences in daily life and a negative impact on their self-image. The findings that the women were striving for independence correspond well with continuity theory [32]. According to this theory, older adults try to maintain continuity of lifestyle by adapting strategies that are connected to their past experiences. The heart of continuity theory is the presumption that people are motivated to continue using adaptive strategies they have developed during adulthood to diagnose situations, and adapt to change. Important elements are idea patterns, lifestyle, personal goals and adaptive capacity, which have a bearing on the outcome [32].

This study showed that women wanted more information, support and practical help from health care professionals regarding, for example, exercising. Unclear, ambiguous and contradictory information from health care professionals leads to uncertainty, for example, about what you can and cannot do, how to exercise, etc., to feel as good as possible. Health care professionals need to be more aware of the multiple levels of change women with vertebral fractures experience in their daily lives and support them in finding successful coping strategies. Information from this study may provide new important knowledge and understanding of the importance for health care professionals to offer empathic and supportive care to women living with prevalent vertebral fracture. One possible effective intervention for the future may be to support women in using self-management strategies so that they can take charge of their situation and positively influence their HRQOL and daily life to achieve independence, but further evaluation is needed.

In a review, the long-term management of the anxiety associated with osteoporosis was shown to include at least three areas of measures: education, exercise, and empowerment [33].

Important aspects of life are described as "managing", "loving" and "having" in this study.

Being independent, retaining the ability to move about freely and managing to live one's life as well as possible despite problems offered hope, satisfaction and self-esteem and thus increased quality of life. The women in this study experienced health when they could take care of themselves, which is consistent with Nordenfelt's holistic theory of health [34], which states that a person is healthy if he/she is in a bodily and mental state that allows him/her to achieve a certain set of goals in life. This set of goals refers to the person's vital goals, i.e. such goals that are necessary and jointly sufficient for the person's long-term happiness [34].

Credibility was addressed through critical judgement, in that all authors took an active part in the data analysis. The analyses were discussed at several meetings, leading to a refinement of the coding process. The authors compared and contrasted the codes, subcategories and categories, and themes with the original text until consensus was reached. Describing the context of the setting, selection and characteristics of the women and using quotations from them to illustrate the findings help the reader to judge the situations, thereby enhancing the transferability of the findings. The sample size of ten women was found to achieve variation, generate information-rich data, and maintain depth in the analysis [24]. There are no rules for sample size in qualitative inquiry approaches; it depends on the purpose of the inquiry [24]. The findings from the study cannot be generalized to all women with vertebral fracture, but the findings may generate hypotheses for further research [35]. The interviewer had experience working as an osteoporosis nurse, which can be seen as a strength as well as a limitation due to preconceptions within the field [36].

The conventional approach in content analysis is limited both in the description of participants' lived experiences and in theory development, because both the sampling and analysis procedures make the theoretical relationship between concepts difficult to assume from the findings. The result of a conventional content analysis is at most a concept development or model building [19].

## Conclusion

This study showed that the women's HRQOL and daily life were strongly affected by the impact of the vertebral fracture. The women in this study are striving for independence or maintaining their independence by trying to manage different types of symptoms and consequences in different ways. One possible effective intervention for the future may be to support women in using self-management strategies so that they can take charge of their situation and positively influence their HRQOL and daily life to achieve independence, but further evaluation is still needed in larger study groups.

## Competing interests

The authors declare that they have no competing interests.

## Authors' contributions

Study design: IH, A-C E, GT, M B-L; data collection: IH; data analysis: IH, A-C E, GT, M B-L; manuscript preparation: IH, A-C E, GT, M B-L. All authors have critiqued revisions of the paper and have approved the final manuscript.

## Authors' information

IH, PhD, RN, Senior lecturer, Department of Medical and Health Sciences, Division of Nursing Science, Faculty of Health Sciences, Linköping University, Department of Endocrinology & Gastroenterology, Osteoporosis Unit, University Hospital, SE-581 85 Linköping, Sweden; A-C E, PhD, RN, Professor, Department of Medical and Health Sciences, Division of Nursing Science, Faculty of Health Sciences, Linköping University, SE-581 85 Linköping, Sweden; GT, PhD, MD, Associate professor, Department of Medical and Health Sciences, Division of Cardiovascular Medicine/Internal Medicine, Faculty of Health Sciences, Linköping University, Department of Endocrinology & Gastroenterology, Osteoporosis Unit, University Hospital, SE-581 85 Linköping, Sweden; M B-L, PhD, RN, Associate professor, Department of Medical and Health Sciences, Division of Nursing Science, Faculty of Health Sciences, Linköping University, SE-581 85 Linköping, Sweden.

## Pre-publication history

The pre-publication history for this paper can be accessed here:

http://www.biomedcentral.com/1472-6955/9/7/prepub
